# Associations between Sugar-Sweetened Beverage Intake and Metabolic Syndrome among Postmenopausal Black Women with Obesity and a Family History of Breast Cancer

**DOI:** 10.1158/2767-9764.CRC-26-0155

**Published:** 2026-06-16

**Authors:** Stacy M. Post, Chiranjeev Dash, Mireille Bright, Jennifer Hicks, Mary Gleason Mills, Lucile L. Adams-Campbell

**Affiliations:** Georgetown Lombardi Comprehensive Cancer Center, Washington, District of Columbia.

## Abstract

Sugar-sweetened beverage (SSB) consumption is linked to increased metabolic syndrome (MetS) and breast cancer risk. This cross-sectional study examined associations between SSB intake and MetS by family history of breast cancer among postmenopausal Black women with obesity. Baseline data of 210 participants from the Focused Intervention on Exercise to Reduce CancEr (FIERCE) clinical trial were used. SSB intake was assessed with the Block 2005 Food Frequency Questionnaire, and family history of breast cancer was collected via a self-report questionnaire. Women with a family history of breast cancer who had high (vs. low) SSB intake had higher triglycerides (29.69 mg/dL higher, *P* = 0.011), lower high-density lipoprotein cholesterol (7.94 mg/dL lower, *P* = 0.027), and a higher MetS *z*-score (1.89 U higher, *P* < 0.001). Adjusting for relevant covariates, greater SSB intake predicted a higher MetS *z*-score (*b* = 0.01, *P*_*interaction*_ = 0.031) and fasting glucose (*b* = 0.02, *P*_*interaction*_ = 0.013) compared with lower SSB intake, only in women with a family history of breast cancer. Greater consumption of SSB subtypes (sugary juices, iced tea, and others) were associated with higher triglycerides, fasting glucose, and/or MetS *z*-score compared with lower consumption, only in women with a family history of breast cancer (*P*_*interactions*_ < 0.05). Higher SSB consumption is linked to higher fasting glucose, triglycerides, and overall MetS risk in postmenopausal Black women with a family history of breast cancer. Targeted multilevel interventions to reduce SSB consumption and improve metabolic health in this population are warranted.

**Significance:**

Higher SSB consumption was linked to higher fasting glucose, triglycerides, and overall MetS risk in postmenopausal Black women with a family history of breast cancer, but not in women without this family history. Findings highlight important heterogeneity in metabolic vulnerability and the need for culturally informed multilevel interventions, such as those targeting healthcare providers and individual decision making, to reduce SSB intake in vulnerable populations with a family history of breast cancer.

## Introduction

Metabolic syndrome (MetS) is characterized by the presence of multiple metabolic abnormalities, including elevated triglycerides, low levels of high-density lipoprotein (HDL), elevated blood glucose, high systolic or diastolic blood pressure, and abdominal obesity (1). One known factor that contributes to MetS is consumption of sugar-sweetened beverages (SSB), such as soda, juice drinks, iced tea, and flavored waters (2). High SSB consumption increases MetS risk by promoting weight gain, insulin resistance, inflammatory biomarkers, and abdominal fat accumulation, which disrupt glucose and lipid metabolism (3).

Findings from the 2003 to 2018 National Health and Nutrition Examination Survey (NHANES) indicate that SSB intake has declined in recent years (4). However, SSB intake among US adults persists at high levels (5), and consumption among Black women, specifically, also remains high. Nearly 80% of Black women with overweight and obesity regularly consume SSBs (6), and non-Hispanic Black women are more likely to consume SSBs compared with non-Hispanic White women (7). Although MetS risk is influenced by factors such as a sedentary lifestyle, low physical activity, and overall dietary patterns (8), high SSB consumption among Black women may contribute to the disproportionate rates of MetS and its components in this population, particularly among middle-aged and older Black women, with 65% of those ages 45 to 65 years meeting the criteria for MetS (9).

### Family history of breast cancer and MetS

Women diagnosed with MetS have a 52% increased risk of developing breast cancer compared with women without this diagnosis, with the strongest associations observed in postmenopausal women (10), and postmenopausal Black women with MetS have a 63% higher risk (11). Because metabolic dysfunction is both a risk factor for breast cancer (12) and a trait that clusters within families (13), women with a family history of breast cancer may be more vulnerable to the adverse effects of SSBs through mechanisms related to both MetS and breast cancer. For example, biological pathways such as variations in DNA and certain genes (e.g., *TCF7L2*) are linked to obesity, insulin signaling, and metabolic inflammation (14)—factors implicated in both MetS and breast cancer risk. In addition, obesity and related cardiometabolic biomarkers are heritable and associated with MetS and breast cell growth and proliferation (12). Thus, women with a family history of breast cancer may be more sensitive to the negative metabolic effects related to high SSB intake relative to women without this family history.

Family history of breast cancer may also reflect environmental factors that contribute to MetS risk and breast cancer predisposition, such as obesogenic environments that cluster within families. Such environments increase the likelihood that children of parents with obesity will develop obesity as adults (15), raise breast cancer risk (16), and may amplify the effect of SSBs on metabolic abnormalities. The confluence of genetic, biological, and environmental factors shared within families may influence both MetS and breast cancer risk, highlighting the need to examine whether dietary factors associated with MetS, such as SSBs, differ by family history of breast cancer.

### Study overview

Research investigating associations between SSB intake and MetS and its components among postmenopausal Black women is limited. Additionally, no research has examined the role of family history of breast cancer in relation to SSB intake on MetS risk. The present study will examine associations between SSB consumption and MetS risk and test whether this relationship varies by family history of breast cancer among postmenopausal Black women who participated in the Focused Intervention on Exercise to Reduce CancEr (FIERCE) trial (17). We hypothesize that among women with (vs. without) a family history of breast cancer, higher SSB consumption will be associated with higher risk of developing MetS and five MetS components (systolic and diastolic blood pressure, HDL cholesterol, fasting glucose, and triglycerides).

## Materials and Methods

### Design and study population

**Figure 1.**
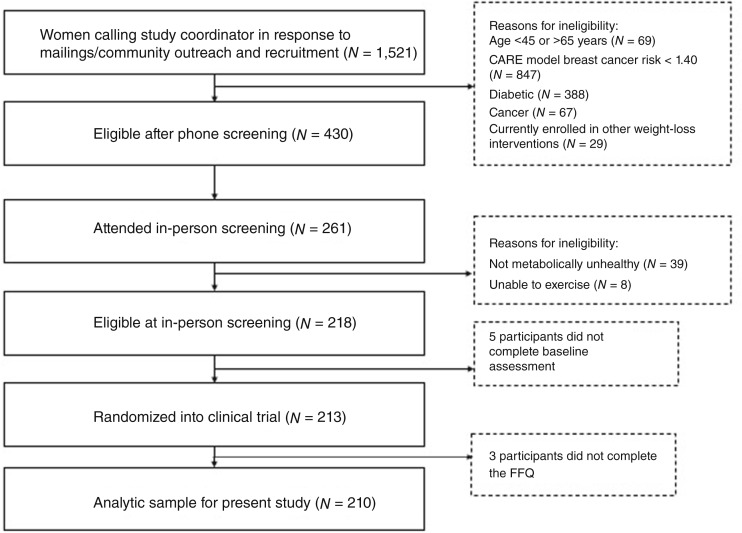
Participant flow diagram for the FIERCE trial and present study analytic sample.

The present study uses data from the FIERCE trial (17), a 6-month, 3-arm randomized controlled trial that evaluated the effects of two exercise interventions versus a control arm on obesity, MetS, and breast cancer biomarkers among postmenopausal Black women at high risk of breast cancer. Details about the FIERCE trial methodology are described in prior work (17, 18). This study includes data from the baseline questionnaire and the Block 2005 Food Frequency Questionnaire (FFQ; ref. 19), which were administered to participants at baseline. The FIERCE trial included 213 physically inactive, postmenopausal Black women between the ages of 45 and 67 years (see Fig. 1). Participants were recruited from majority African-American communities in the Washington, DC, metropolitan area. All participants were physically inactive, had increased risk of breast cancer, abdominal obesity, and at least one other MetS component at baseline (17). Exclusion criteria for the trial included a history of cancer (except nonmelanoma skin cancer), diabetes, low projected breast cancer risk score, current regular exercise, enrollment in another trial or weight-loss program, or inability to adhere to the intervention schedule. Three participants did not complete the FFQ and thus were omitted. All participants provided written informed consent. The study protocol was approved by the Georgetown University–MedStar Health Institutional Review Board IRB #2012-012.

### Measures

#### Metabolic variables

According to the US National Cholesterol Education Program Adult Treatment Panel III (ATP-III) definition, criteria for MetS includes having three or more of the following components: waist circumference ≥88 cm, fasting glucose ≥100 mg/dL, systolic blood pressure ≥130 or ≥85 mm Hg diastolic blood pressure, triglycerides ≥150 mg/dL, and HDL cholesterol <50 mg/dL (20). Systolic and diastolic blood pressure levels were assessed as the average of measurements taken after 10 and 20 minutes of seated rest. Fasting glucose, triglycerides, and HDL cholesterol were obtained from fasting morning venous blood samples. Waist circumference was measured with a measuring tape around the navel and reported in centimeters.

MetS was examined continuously using a MetS *z*-score, with higher scores indicating higher risk or severity of MetS. The MetS *z*-score variable was calculated using the following formula per ATP-III criteria, using the standard deviations (SD) for each MetS component in the FIERCE trial baseline sample: [(waist circumference – 88)/13.77] + [(fasting blood glucose – 100)/13.09] + [(mean arterial pressure – 100)/11.29] + [(triglycerides – 150)/55.08] + [(50 – HDL)/16.83] (17). Mean arterial pressure was calculated as [((systolic blood pressure) + (diastolic blood pressure × 2))/3]. MetS was also examined as a dichotomized variable to indicate whether participants had three or more MetS components. The five MetS components of interest were examined continuously and as dichotomized variables, based on ATP-III cutoff values, to indicate high systolic or diastolic blood pressure, high fasting glucose, high triglycerides, and low HDL.

#### SSB intake

SSB intake was measured using the Block 2005 FFQ, a dietary assessment tool validated for use with Black women (21). Participants reported the frequency and quantity of consumption of five types of SSBs “in the past year or so,” including sugary juice/punch (such as Hi-C, cranberry juice cocktail, Hawaiian punch, and Tang), drinks with some juice (such as Sunny Delight), iced tea (homemade, instant, or bottled such as Nestea, Lipton, Snapple, and Tazo), soft drinks (such as cola, Sprite, or orange soda), and “other” SSBs (such as Kool-Aid, lemonade, sports drinks like Gatorade, or fruit flavored drinks). Participants who reported drinking soft drinks or iced tea specified whether the beverages were regular, diet, or low-calorie. Frequency was reported as 1 (*never*), 2 (*a few times per year*), 3 (*once per month*), 4 (*2–3 times per month*), 5 (*once per week*), 6 (*2 times per week*), 7 (*3–4 times per week*), 8 (*5–6 times per week*), or 9 (*every day*). Participants then reported their typical quantity on the days SSBs were consumed. For sugary juice/punch, drinks with some juice, and iced tea, participants reported consumption in glasses (1/2, 1, 2, or 3). Soft drink intake was reported in cans or bottles (1, 2, or 3 cans, or one 20-ounce bottle). Intake of beverages categorized as “other” (e.g., Kool-Aid and sports drinks) was reported in glasses or 20-ounce bottles (1 or 2 glasses or bottles). Total intake of each subtype was computed by multiplying the frequency × quantity variables. Consumption of diet or low-calorie soft drinks or iced tea was recoded as “never” for quantity.

Overall SSB consumption was classified as daily absolute intake (g/day). To account for participants’ differences in total food energy intake, daily absolute intake of SSBs in grams was standardized using the following formula: (average daily intake of SSBs in grams/average total daily food energy intake in kilocalories) × 1,000. Total kilocalories per day from SSBs and percentage of average daily kilocalories from added sugar (e.g., sweets and desserts) were also collected via the FFQ. The percentage of daily calories from added sugar was dichotomized to reflect whether participants met US dietary guidelines (0 = less than 10% of total daily calories, 1 = 10% or greater; ref. 22).

FFQs were scanned and analyzed by Block Dietary Data Systems using a nutrient content database via the US Department of Agriculture Nutrient Database for Standard Reference and national food consumption data [the Third NHANES (NHANES III) and the Continuing Survey of Food Intakes by Individuals].

#### Family history of breast cancer

Participants self-reported how many first-degree relatives (mother, full-blooded sisters, or daughters) ever had breast cancer (0, 1, >1). This variable was dichotomized to indicate the absence (0 = no relatives) or presence (1 = 1 or more relatives) of family history of breast cancer.

#### Sociodemographics

Age, marital status, education, and income were collected via the baseline questionnaire to characterize the study participants. Participants also reported whether they currently smoke, previously smoked, or never smoked; responses were dichotomized as never smoker versus those who currently or previously smoked.

#### Anthropometrics

The study team collected height and weight measurements, which were used to calculate body mass index (BMI; kg/m^2^).

#### Covariates

Age, smoking status, and income were included as covariates in analyses. Average daily consumption of alcohol (standard drinks per day), red meat in ounces, and saturated fat in grams were also measured via the FFQ, standardized, and included as covariates due to their associations with MetS and its components (23–25). Physical inactivity was an eligibility criterion; therefore, physical activity was not included as a covariate due to limited variability.

### Statistical analyses

All analyses were conducted using data collected from questionnaires administered at baseline. Frequencies and descriptive statistics (means and SDs) were used to characterize participants’ sociodemographics. Independent samples *t* tests examined differences in sociodemographics, family history of breast cancer, added sugar consumption, and presence or absence of MetS by high versus low SSB intake (categorized as above or below the mean of total energy-adjusted SSB intake).

*χ* ^2^ and independent samples *t* tests examined whether there were significant differences in consumption of SSB subtypes, total energy-adjusted SSB intake, kilocalories per day from SSBs, and prevalence of any daily SSB intake between participants with and without a family history of breast cancer. Independent samples *t* tests examined whether there were significant differences in MetS *z*-score and continuous MetS components between participants with high versus low SSB intake, stratified by family history of breast cancer.

The primary outcome was MetS *z*-score in regression models. Associations with the presence or absence of MetS and continuous and dichotomized MetS components were examined as secondary analyses. Multivariable linear regression models assessed the overall association between SSB intake (total energy-adjusted and five SSB subtypes) and MetS *z*-score and continuous MetS components. Hayes (26) PROCESS macro, model 1, tested whether associations between SSB intake and MetS *z*-score and continuous MetS components were modified by family history of breast cancer. Multivariable logistic regressions tested associations between high versus low SSB intake and the presence or absence of MetS and dichotomized MetS components. Hayes’ PROCESS macro, model 1, tested whether associations between high versus low SSB intake and categorical MetS outcomes were modified by family history of breast cancer. Models with observations showing a Cook’s distance ≥0.5 were reestimated after excluding these outliers. Seventeen participants had inadequate samples for HDL cholesterol and triglycerides, and thus sample mean values were imputed. Significance was set at *P* < 0.05. All analyses were conducted using SPSS statistical software (version 30; RRID: SCR_002865).

## Results

### Study population characteristics

The study population includes a total *N* = 210 with a mean age of 57.76 (*SD* = 5.01) and BMI of 35.86 (*SD* = 6.94). Characteristics of study participants grouped by family history of breast cancer are shown in Table 1. Forty percent of participants had a family history of breast cancer, and 60% had no family history. The majority of participants had more than a high school education, reported a marital status other than married/living with partner, and identified as a current or former smoker. In addition, 77.14% of participants met the criteria for MetS, and 75.24% consumed ≥10% of total daily calories from added sugar. Among the most prevalent SSB subtypes, 86.67%, 83.80%, and 66.20% of participants consumed iced tea, soft drinks, and sugary juice/punch, respectively, with no significant differences based on family history of breast cancer (see Table 2). The average daily SSB intake was 155.5 g per 1,000 kcal, and all but 13 participants (93.8%) reported daily consumption of SSBs.

**Table 1.** Baseline characteristics by family history of breast cancer in the FIERCE study population of postmenopausal Black women.

Variable	Total (*N* = 210)	Family history of breast cancer (*N* = 84)	No family history of breast cancer (*N* = 126)
	*N* (%)	*N* (%)	*N* (%)
Smoking status	​	​	​
Current/former	99 (47.14%)	43 (20.48%)	56 (26.67%)
Never	111 (52.86)	41 (19.52%)	69 (32.86%)
Education	​	​	​
Less than a high school education	16 (7.62%)	11 (5.24%)	5 (2.38%)
High school diploma	18 (8.57%)	10 (4.76%)	8 (3.81%)
More than a high school education	175 (83.33%)	73 (34.76%)	112 (53.33%)
Not reported	1 (0.48%)	0 (0%)	1 (0.48%)
Marital status	​	​	​
Married/living with partner	55 (26.19%)	17 (8.10%)	38 (18.10%)
Single/divorced/separated/widowed	154 (73.33%)	67 (31.90%)	87 (41.43%)
Not reported	1 (0.48%)	0 (0%)	1 (0.48%)
SSB intake	​	​	​
Any daily intake	197 (93.81%)	80 (38.10%)	117 (55.71%)
No daily intake	13 (6.19%)	4 (1.90%)	9 (4.29%)
Added sugar (all added sugars including SSBs)	​	​	​
<10% of total calories	52 (24.76%)	24 (11.43%)	28 (13.33%)
≥10% of total calories	158 (75.24%)	60 (28.57%)	98 (46.67%)
MetS	​	​	​
Meets criteria for MetS	162 (77.14%)	17 (8.10%)	95 (45.24%)
Does not meet criteria for MetS	48 (22.86%)	67 (31.90%)	31 (14.76%)

​	Mean (SD)	Mean (SD)	Mean (SD)
Age	57.76 (5.01)	54.85 (5.57)	59.70 (3.45)
BMI (kg/m^2^)	35.86 (6.94)	36.88 (7.11)	35.19 (6.78)

**Table 2.** Prevalence of any daily SSB intake, stratified by family history of breast cancer, in the FIERCE study population of postmenopausal Black women.

SSB	Total population (*N* = 210)	Family history of breast cancer (*N* = 84)	No family history of breast cancer (*N* = 126)	Between-group *P* value
	%, M (SD)	%, M (SD)	%, M (SD)	
Sugary juice/punch	66.20%	63.10%	68.25%	0.51
Drinks with some juice	40.50%	42.86%	38.89%	0.56
Iced tea	86.67%	82.14%	89.68%	0.12
“Other” SSBs (e.g., Kool-Aid and sports drinks)	60.90%	59.52%	61.90%	0.62
Soft drinks	83.80%	88.10%	80.95%	0.17
Average daily intake of SSB, grams	155.45 (173.6)	158.85 (171.49)	153.18 (175.64)	0.82
Kilocalories per day from SSBs	116.69 (155.53)	124.29 (168.43)	111.63 (146.78)	0.58
Reported any daily SSB intake	93.81%	95.24%	92.06%	0.47

Percentages indicate the share of participants reporting any intake of each SSB subtype.

### Associations between SSB intake and MetS risk

**Figure 2.**
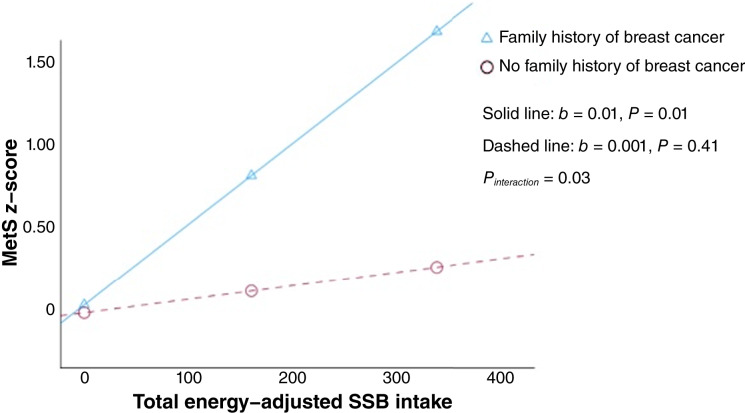
Interaction between total energy-adjusted SSB intake and family history of breast cancer on MetS *z*-score in the FIERCE study population of postmenopausal Black women. Total energy-adjusted SSB intake was standardized using the following formula: (average daily intake of SSB in grams/average total daily food energy intake in kilocalories) × 1,000. The model was adjusted for age, smoking status, income, and dietary factors (alcohol, red meat, and saturated fat intake).

Multivariable linear regressions testing the influence of family history of breast cancer demonstrated that higher total energy-adjusted SSB intake was significantly associated with a higher MetS *z*-score and fasting glucose in women with a family history of breast cancer but not in women without this family history. Corresponding interaction terms were significant for both fasting glucose (*P* = 0.01) and MetS *z*-score (*P* = 0.03; see Fig. 2). Sugary juice/punch intake was significantly associated with higher triglycerides, and MetS *z*-score only in women with a family history of breast cancer (see Table 3). Significant interactions between family history of breast cancer and sugary juice/punch intake were observed for fasting glucose (*P* = 0.03), triglycerides (*P* = 0.03), and MetS *z*-score (*P* < 0.001). In addition, only in women with a family history of breast cancer, higher intake of iced tea and drinks with some juice was significantly associated with higher triglycerides. Among women with a family history of breast cancer, higher iced tea intake was also associated with MetS *z*-score, with a corresponding significant interaction (*P* = 0.04). Similarly, higher intake of “other” SSBs (e.g., Kool-Aid, sports drinks) was significantly associated with higher fasting glucose and triglycerides in women with a family history of breast cancer, with significant interactions for fasting glucose (*P* = 0.01) and MetS *z*-score (*P* = 0.02) but not triglycerides (*P* = 0.10). Soft drink intake showed no significant associations or interaction effects with continuous MetS outcomes. Similarly, consumption of SSB subtypes was not significantly associated with systolic or diastolic blood pressure or HDL cholesterol in women with or without a family history of breast cancer, and no interaction terms for these components were significant (*P*s > 0.05).

**Table 3.** Multivariable-adjusted associations of SSB intake and SSB subtypes with continuous MetS outcomes by family history of breast cancer in the FIERCE study population of postmenopausal Black women.

​	Family history of breast cancer (*N* = 84)			No family history of breast cancer (*N* = 126)	
	Unstandardized B	Std. error	Unstandardized B	Std. error	Interaction *P* value
All SSBs	​	​	​	​	​
Systolic blood pressure (mm Hg)	0.01	0.01	0.004	0.01	0.29
Diastolic blood pressure (mm Hg)	0.01	0	0.001	0.01	0.08
Fasting glucose (mg/dL)	0.02*	0	−0.01	0.01	0.01*
Triglycerides (mg/dL)	0.06	0.03	0.05	0.03	0.88
HDL cholesterol (mg/dL)	−0.02	0.01	−0.01	0.01	0.18
MetS *z*-score	0.01**	0.002	0.001	0.001	0.03*
Sugary juice/punch	​	​	​	​	​
Systolic blood pressure (mm Hg)	0.02	0.34	0.11	0.26	0.92
Diastolic blood pressure (mm Hg)	0.13	0.21	0.09	0.16	0.63
Fasting glucose (mg/dL)	0.36	0.27	−0.32	0.19	0.03*
Triglycerides (mg/dL)	3.84***	1.07	0.53	0.85	0.03*
HDL cholesterol (mg/dL)	−0.44	0.33	0.15	0.25	0.17
MetS *z*-score	0.21***	0.05	−0.02	0.04	<0.001***
Drinks with some juice	​	​	​	​	​
Systolic blood pressure (mm Hg)	−0.78	0.44	−0.20	0.41	0.47
Diastolic blood pressure (mm Hg)	−0.21	0.29	0.02	0.27	0.82
Fasting glucose (mg/dL)	0.44	0.36	−0.50	0.30	0.07
Triglycerides (mg/dL)	3.43*	1.45	−0.05	1.37	0.1
HDL cholesterol (mg/dL)	0.04	0.44	0.13	0.38	0.79
MetS *z*-score	0.08	0.07	−0.07	0.06	0.08
Iced tea	​	​	​	​	​
Systolic blood pressure (mm Hg)	0.03	0.28	0.07	0.21	0.81
Diastolic blood pressure (mm Hg)	0.22	0.18	0.12	0.14	0.43
Fasting glucose (mg/dL)	0.41	0.23	−0.11	0.16	0.06
Triglycerides (mg/dL)	2.07*	0.93	1.32	0.70	0.62
HDL cholesterol (mg/dL)	−0.39	0.27	−0.22	0.21	0.51
MetS *z*-score	0.14**	0.04	0.05	0.03	0.04
Soft drinks	​	​	​	​	​
Systolic blood pressure (mm Hg)	0.06	0.22	0.11	0.25	0.71
Diastolic blood pressure (mm Hg)	−0.01	0.22	−0.06	0.17	0.58
Fasting glucose (mg/dL)	0.27	0.18	−0.22	0.18	0.08
Triglycerides (mg/dL)	0.46	0.72	0.87	0.81	0.62
HDL cholesterol (mg/dL)	0.30	0.33	0.22	0.27	0.87
MetS *z*-score	0.05	0.04	0.06	0.03	0.7
“Other” SSBs (e.g., Kool-Aid and sports drinks)	​	​	​	​	​
Systolic blood pressure (mm Hg)	−0.30	0.34	0.06	0.27	0.49
Diastolic blood pressure (mm Hg)	−0.01	0.22	−0.07	0.17	0.75
Fasting glucose (mg/dL)	0.62*	0.27	−0.34	0.19	0.01*
Triglycerides (mg/dL)	2.43*	1.11	−0.09	0.9	0.10
HDL cholesterol (mg/dL)	0.29	0.33	0.22	0.27	0.85
MetS *z*-score	0.09	0.06	−0.06	0.04	0.02*

For stratified analyses, * indicates *P* < 0.05; ** indicates *P* < 0.01; and *** indicates *P* < 0.001. Models were adjusted for age, smoking status, income, and dietary factors (alcohol, red meat, and saturated fat intake).

The unadjusted mean differences in MetS *z*-score and MetS components between high versus low SSB intake, stratified by family history of breast cancer, are presented in Table 4. Women with a family history who had high (vs. low) SSB intake had significantly higher triglycerides (29.69 mg/dL higher; *P* = 0.011), lower HDL cholesterol (7.94 mg/dL lower; *P* = 0.03), and higher MetS *z*-score (1.89 U higher; *P* < 0.001). There were no significant differences in fasting glucose or diastolic and systolic blood pressure between high versus low SSB intake among women with a family history of breast cancer. There were also no significant differences in MetS *z*-score or any components between high versus low SSB intake among women *without* a family history of breast cancer.

**Table 4.** Differences in MetS *z*-score and continuous MetS components by high vs. low SSB intake, stratified by family history of breast cancer in the FIERCE study population of postmenopausal Black women.

MetS component	Total (*N* = 84)	Family history of breast cancer (*N* = 84)				Total (*N* = 126)	No family history of breast cancer (*N* = 126)			
		Low SSB intake (*N* = 49)	High SSB intake (*N* = 35)	Mean difference	Between-group *P* value		Low SSB intake (*N* = 80)	High SSB intake (*N* = 46)	Mean difference	Between-group *P* value
		Mean (SD)	Mean (SD)				Mean (SD)	Mean (SD)		
Systolic blood pressure (mm Hg)^a^	129 (16)	128 (15)	130 (18)	−2	0.73	134 (18)	134 (18)	132 (17)	2	0.57
Diastolic blood pressure (mm Hg)^a^	82 (11)	80 (10)	85 (10)	−5	0.08	82 (11)	82 (11)	82 (11)	0	0.83
Fasting glucose (mg/dL)	105 (13.39)	104.29 (13.09)	106 (13.94)	−1.71	0.57	101.98 (12.80)	102.61 (12.71)	100.87 (13.03)	1.74	0.46
Triglycerides (mg/dL)	109.45 (53.21)	97.08 (42.78)	126.77 (61.63)	−29.69	0.01*	107.01 (56.47)	105.17 (56.58)	110.19 (56.76)	−5.02	0.63
HDL cholesterol (mg/dL)	56.77 (16.36)	60.08 (18.74)	52.13 (10.94)	7.94	0.03*	61.07 (16.97)	62.13 (18.41)	59.24 (14.14)	2.88	0.36
MetS *z*-score	0.78 (2.72)	−0.01 (2.66)	1.88 (2.45)	−1.89	<0.001*	0.07 (2.44)	−0.001 (2.33)	0.19 (2.66)	−0.19	0.68

a Values are rounded to the nearest integer for consistency with clinical reporting.

*Indicates statistical significance.

Multivariable logistic regressions testing the influence of family history of breast cancer demonstrated that there were no significant associations between total energy-adjusted SSB intake and the presence or absence of MetS or dichotomized MetS components in women with or without a family history of breast cancer (*P*s > 0.21). In women with a family history of breast cancer, higher intake of soft drinks was associated with 72.2% lower odds of having systolic blood pressure ≥130 mm Hg. In addition, in women without a family history of breast cancer, higher intake of “other” SSBs (e.g., Kool-Aid and sports drinks) was associated with 74.6% lower odds of having a fasting glucose level of ≥100 mg/dL. However, the number of participants classified as having a family history of breast cancer and high systolic blood pressure or fasting glucose were 8 and 7, respectively, and thus the small number of cases in these cells likely inflated the odds ratios. There were no significant associations between any other SSB subtype and dichotomized MetS outcomes in women with or without a family history of breast cancer and no significant interactions (*P*s > 0.11).

Multivariable linear regressions across the aggregate study population demonstrated that there was a significant association between total energy-adjusted SSB intake and MetS *z*-score (*b* = 0.003, *P* = 0.01) but no significant associations with any MetS component (*P*s > 0.07). Higher intake of sugary juice/punch (*b* = 1.46, *P* = 0.03) and iced tea (*b* = 1.13, *P* = 0.04) was significantly associated with higher triglycerides, and higher iced tea intake was also associated with a higher MetS *z*-score (*b* = 0.08, *P* = 0.003). There were no other significant associations between SSB subtypes and MetS *z*-score or continuous components (*P*s > 0.08). Lastly, multivariable logistic regressions across the study population demonstrated that high (vs. low) SSB intake had no significant associations with the presence or absence of MetS or dichotomized MetS components (all *P*s > 0.12).

## Discussion

This study demonstrated that, only among women with a family history of breast cancer, higher total energy-adjusted SSB intake and consumption of sugary juice/punch, iced tea, and other SSBs (e.g., Kool-Aid, sports drinks, and lemonade, but not soft drinks) were linked with one or more adverse metabolic outcomes, including higher triglycerides, fasting glucose, and MetS risk. Associations were most apparent when MetS outcomes were examined continuously, suggesting that the MetS *z*-score and continuous components allowed for great sensitivity to detect incremental subclinical metabolic changes. Notably, unadjusted analyses observed differences in HDL cholesterol and triglycerides in fasting glucose between high and low SSB intake in women with a family history of breast cancer. The results suggest that consuming SSBs may have meaningful effects on fasting glucose and triglyceride levels in postmenopausal Black women with obesity who have a family history of breast cancer, which seems to heighten overall MetS risk. Findings also underscore that Black women with obesity are metabolically heterogenous and that negative dietary exposures may have a more pronounced impact on some subgroups, such as those with a family history of breast cancer.

The present findings are consistent with prior research demonstrating that SSB consumption is associated with higher triglycerides (27, 28), fasting glucose (29, 30), and MetS risk (3). However, although unadjusted analyses observed an association between high (vs. low) SSB intake and HDL cholesterol in women with a family history of breast cancer, likely due to lack of control for other dietary factors (e.g., alcohol and red meat intake), multivariable-adjusted analyses did not show an association, contrasting prior research showing an inverse association (27, 31). The results also contrast previous work demonstrating that high SSB intake is associated with increased diastolic and systolic blood pressure levels. For example, previous research documented that SSB intake was associated with a 26% higher risk of hypertension and a 0.24 mm Hg increase in systolic blood pressure per 250 g increase of SSBs (32). In addition, we did not observe an association between soft drink intake and MetS or its components, contrasting longitudinal and review studies showing consumption of sugar-sweetened soft drinks predicts increased MetS risk and its components (33, 34). Variability in observed associations between SSB intake, including soft drinks, HDL cholesterol, and blood pressure, may reflect differences in study design, covariate adjustment, or population characteristics, as limited research has explored these relationships among Black women specifically.

This is the first study to report that having a family history of breast cancer may heighten metabolic vulnerability to dietary exposures in postmenopausal Black women. Dash and colleagues (17) found that postmenopausal Black women with family history of breast cancer who participated in an exercise intervention arm (vs. control arm) during the FIERCE trial had improved metabolic profiles between a baseline assessment and 6-month follow-up. The improved metabolic effects driven by exercise in Dash and colleagues (17) and the positive association between SSB intake and MetS risk in the present study suggest that women with a family history of breast cancer may show greater metabolic responsiveness to positive behaviors (like exercise) and more metabolic vulnerability to negative exposures (like SSBs) through shared biological and environmental pathways related to both breast cancer risk and metabolic health. Individuals with a family history of breast cancer may be more susceptible to inherited variations in these shared pathways, such as those relating to insulin, insulin-like growth factor-1 (IGF-1), and estrogen (35, 36), potentially modifying how SSB consumption affects insulin resistance, obesity, and metabolic abnormalities. In addition, genetic variations linked to obesity and insulin signaling may also interact with lifestyle factors often modeled within families, such as diet, to shape risk for metabolic dysfunction and breast cancer. For example, the combination of alcohol consumption, BMI, and genetic variants related to IGF-I/insulin phenotypes—factors associated with metabolic vulnerabilities (37, 38)—increased the risk of postmenopausal breast cancer (39). Other lifestyle and environmental factors that cluster within families, such as physical activity (40) and exposure to endocrine disruptors linked to both cancer and metabolic health (e.g., perfluoroalkyl substances and phthalates; ref. 41), may influence both MetS and breast cancer risk. Thus, investigating how shared biological and environmental mechanisms linking dietary exposures, such as SSBs, with MetS in women with a family history of breast cancer is an important next step.

Average SSB intake was 155.45 g per 1,000 kcal, which is equivalent to about one can of soda per day on a 2,000-calorie diet. This level of SSB consumption is consistent with the national average for US adults who drink SSBs on a given day (5). However, the proportion of participants reporting *any* daily SSB consumption was substantially higher than national averages: 93.8% in this study population, compared with 49% among US adults (15) and 74.8% among Black adults (4). Additionally, the proportion of study participants who met US dietary guidelines for added sugar was substantially lower than national averages: only 24.8% of participants consumed <10% of total daily calories from added sugar compared with 42% of US adults and 34% of Black adults (42). Such nearly universal daily SSB consumption and low adherence to dietary guidelines suggest that this population of metabolically unhealthy postmenopausal Black women may have consistent high exposure to SSBs.

Chronic exposure to SSBs likely reflects a context shaped by environmental and socioeconomic factors. Although participants had a range of socioeconomic levels, the median household income was $35,000 to $49,000, which is well below the median US household income of nearly $84,000 (43). Lower-income households are more likely to consume SSBs (5), reside in “food deserts” (44), have higher obesity prevalence (44), and experience greater breast cancer incidence and mortality rates than higher-income households (45). Additionally, food marketing advertisements disproportionately target lower-income neighborhoods with a high density of African-American residents, negatively affecting the diet quality of these populations (46). Thus, although study findings suggest that individual-level behavioral interventions aimed at reducing SSB consumption among postmenopausal Black women with a family history of breast cancer are necessary, further research examining the social determinants that influence SSB intake in this population, including socioeconomic level, food insecurity, and neighborhood disadvantage, remains critical.

### Strengths and limitations

Strengths of this study include a unique study population of Black women who are traditionally underrepresented in medical research. Additionally, assessing MetS components with biomarker measurements at baseline provided objective indicators of metabolic health and increased internal validity. Finally, examining SSB intake and MetS risk within the context of family history of breast cancer adds novel insights into possible shared mechanisms linking SSB intake with both MetS and breast cancer risk.

This study had several limitations worth noting. Although the FFQ is a validated dietary assessment instrument, food energy intake is often underreported, leading to inaccurate reporting (47). To address this, absolute quantity of SSB intake was standardized into grams of SSBs per 1,000 kcal to adjust for within-person bias and reduce measurement error. Furthermore, this sample represented metabolic unhealthy Black women at high projected risk of breast cancer, and the results might not be generalizable to more physically active or metabolically healthy postmenopausal Black women and other populations. Finally, the cross-sectional design of this study cannot determine a causal relationship between SSB intake and MetS, and thus prospective studies are needed.

### Conclusion

This analysis of clinical trial baseline data demonstrated that SSB consumption predicted higher triglycerides, fasting glucose, and MetS risk in postmenopausal Black women with a family history of breast cancer but not in those without this family history. Additionally, self-reported SSB consumption was nearly universal among participants. These findings highlight the need for culturally informed multilevel interventions, such as those targeting healthcare providers and individual decision making, to reduce SSB intake in high-risk populations. Structural interventions and food policy changes may be other critical factors in reducing SSB intake among minoritized populations with a family history of breast cancer. The increasing rates of MetS and obesity in postmenopausal Black women suggest that it is necessary to further understand specific dietary factors prevalent in this population to develop effective interventions aimed at improving metabolic health and reducing breast cancer risk.

## Data Availability

The data generated in this study are available upon request from the corresponding author.
